# Facing up to Problem Gambling: Tracing the Emergence of Facial Recognition Technology as a means of Enforcing Voluntary Self-Exclusion

**DOI:** 10.1007/s10899-024-10308-4

**Published:** 2024-05-09

**Authors:** Neil Selwyn, Gavin Smith, Mark Andrejevic, Xin Gu, Chris O’Neill

**Affiliations:** 1 School of Education, Culture & Society, Faculty of Education, Monash University, Melbourne, Australia; 2 School of Sociology, College of Arts & Social Sciences, Australian National University, Canberra, Australia; 3 School of Media & Communication, Faculty of Arts, Monash University, Melbourne, Australia; 4 Alfred Deakin Institute for Citizenship and Globalization, Deakin University, Melbourne, Australia

**Keywords:** Problem gambling, Self-exclusion, Facial recognition, Computers

## Abstract

Computer technology has long been touted as a means of increasing the effectiveness of voluntary self-exclusion schemes – especially in terms of relieving gaming venue staff of the task of manually identifying and verifying the status of new customers. This paper reports on the government-led implementation of facial recognition technology as part of an automated self-exclusion program in the city of Adelaide in South Australia—one of the first jurisdiction-wide enforcements of this controversial technology in small venue gambling. Drawing on stakeholder interviews, site visits and documentary analysis over a two year period, the paper contrasts initial claims that facial recognition offered a straightforward and benign improvement to the efficiency of the city’s long-running self-excluded gambler program, with subsequent concerns that the new technology was associated with heightened inconsistencies, inefficiencies and uncertainties. As such, the paper contends that regardless of the enthusiasms of government, tech industry and gaming lobby, facial recognition does not offer a ready ‘technical fix’ to problem gambling. The South Australian case illustrates how this technology does not appear to better address the core issues underpinning problem gambling, and/or substantially improve conditions for problem gamblers to refrain from gambling. As such, it is concluded that the gambling sector needs to pay close attention to the practical outcomes arising from initial cases such as this, and resist industry pressures for the wider replication of this technology in other jurisdictions.

## Introduction

Australia has some of the highest rates of gambling in the world, with over 80 percent of adults reporting that they engage in at least one form of gambling on an annual basis. Particularly prevalent is gambling through electronic slot machines – referred to in Australia as ‘pokies’ (shorthand for poker machines). Around 4 percent of Australians report gambling through these machines on a weekly basis, with playing the pokies estimated to account for over 60 percent of local annual gambling spend. As Drew Rooke (2018, n.p) describes:

“Australia’s poker machines are one of the most intense forms of gambling available in the world, radically different from the fruit machines offered in pubs in the United Kingdom, or the pachinko machines in Japan. [Machines] can be played once every couple of seconds, offer enormous jackpots reaching into the tens of thousands of dollars ... If played at their maximum bet and maximum speed, they can easily consume $600 to $1200 in an hour”.

These high levels of gambling have understandably prompted growing concerns in Australia over gambling as a public health issue. Around 1.2 percent of the adult population is estimated to experience what can be classed as ‘problem gambling’ – i.e. repetitive gambling behaviour that persists despite harmful and negative consequences. Against this background, Australian policymakers and the local gaming industry have long worked to address problem gambling, with self-exclusion schemes running since the 1990s. Also known as ‘self-barring’, such schemes operate to “allo[w] patrons to ban themselves from a venue or its gaming facilities for a specified period of time” (Hing & Nuske, 2012, p.457). Schemes usually operate on a voluntary basis, with self-excluded individuals agreeing that gaming venue staff at specified locations have authorisation to deny them access for a specified period of time. Self-exclusion agreements usually place responsibility for compliance on the individual, who will be removed from any gaming venue they attempt to access as well as being potentially charged with trespass depending on the particular jurisdiction (Gainsbury, 2014). Individuals agree to their personal details and photographic headshots being distributed to all relevant venues, and waive the right to sue nominated venues or venue staff in the event of any transgression.

Traditionally these self-exclusion schemes have been found to be of limited effectiveness. On one hand, self-exclusion programs do allow individuals to make a public commitment to abstain from gambling. For some, this act of acknowledging and pledging can be therapeutic, making themselves and what they do visible to different audiences (e.g. state officials, venue staff, family and friends), and can function as one of many levers to better govern and reduce their harmful behaviour. Thus, while only a small proportion of problem gamblers enrol in these programs, the potential embarrassment of being caught publicly if contravening the agreement does act as a clear deterrent for some. On the other hand, self-exclusion programs in their current manifestation suffer from a number of well-noted shortcomings. These include the difficulty for staff in busy venues to effectively monitor for breaches, limits to the number of venues that any exclusion order can cover, and the possible conflict of interest for venues in enforcing a program that might impact on their income (Hing & Nuske, 2012; Pickering & Blaszczynski, 2022).

## Facial Recognition as a means of Enforcing Gambling Self-Exclusion Programs

This paper examines the recent coming-together of gambling self-exclusion schemes in Australia and facial recognition technology – offering what some commentators see as a high-tech, automated enhancement to the basic premise of gambling self-exclusion. In broad terms, facial recognition is computer-based technology that can detect and extract a human face from a digital image, and then match this face against a database of pre-identified faces. The development of facial recognition stretches back to the 1960s, but has progressed rapidly over the past five years or so due to general advances in computational processing power and increased data storage capabilities required to develop and train large-scale machine learning models. Another important development has been advances in cheap and powerful digital camera hardware, with the past few years seeing high definition cameras being installed throughout everyday public contexts, as well as integrated into all manner of objects and personal devices – from smartphones to supermarket checkouts. Now, it is possible for basic facial recognition technology to be installed for relatively little cost, with systems being integrated into existing CCTV systems in venues such as convenience stores, banks, schools, workplaces and people’s homes (see Selwyn et al., 2024). Moreover, public attitudes are progressively reported to be becoming more receptive to the application of FRT in contexts where it is seen to be social desirable to be reducing risk and/or harm (Kostka et al., 2023).

Against this background, gambling and gaming venues are increasingly being promoted by industry and regulatory authorities as viable sites for the implementation of FRT. In keeping with their pioneering role in consumer surveillance technology, large casinos have been enthusiastic ‘early adopters’ of facial recognition well before the technology was considered reliable enough to be used elsewhere in society. Bally's Las Vegas casino is generally acknowledged as the first venue to implement a rudimentary facial recognition system as far back as 1994. By the mid-2000s, around 160 North American casinos were accessing the ‘Surveillance Information Network’ based on a shared photographic database of over 2500 individuals deemed as known ‘casino threats’ which could be run through each venue’s facial recognition system (Norris, 2019). Now, most large casinos operate some form of facial recognition technology, and in a few jurisdictions are legally required to do so in order to bar entry of under-age patrons and to maintain self-exclusion lists. All told, keeping tabs on who is entering the premises and monitoring their movements is a familiar element of day-to-day operations within large gaming venues, with many now boasting expensive high-specification facial recognition systems to perform this form of exclusion enforcement.

The focus for this paper, however, is the recent incursion of facial recognition into small gaming venues in Australia as part of modernising self-exclusion schemes. Facial recognition technology has been discussed and piloted since the 2000s as a potential means of enforcing self-exclusion programs from gaming venues. On one hand, there has been steady support over the past decade for facial recognition being added to self-exclusion schemes. Indeed, critics of manually-enforced paper-based exclusion schemes have long endorsed exploring the possibility of “computerised identification checks for enforcement of self-exclusion” (Gainsbury, 2014, p.229). Conversely, surveys stretching back to the mid 2000s find majority support amongst self-excluded gamblers for FRT as a tool to better identify and deter them at venue entrances (Verlik, 2008, in Gainsbury, 2014).

Yet, while acknowledged as offering a promising alternative to requiring venue staff to memorise photos in order to identify self-excluded gamblers, the technology was still considered ‘unrealistic’ up until a few years ago. As one review concluded in the mid-2010s, “while facial recognition software may have some effectiveness in identifying self-excluded gamblers, it is not clear that the technology is a best practice” (Miller, 2015, p.41). Nevertheless, rapid technical advances in FRT over the past five years or so are now prompting a wider take-up of the technology amongst smaller gaming venues, with pressure for adoption, implementation and usage coming from various sources and agents, vendors of the technology, specific venues, state regulators of the gaming industry, and some self-excluded individuals and their families. As the 2020s progress, FRT is beginning to be seen as an affordable and reliable means of overcoming the inconsistencies of manually-enacted identification and verification processes. All told, the development of FRT over the past five years or so has led to the point where these initial discussions and recommendations have now reached a point of realisation.

## Research Questions

In this paper we report on the state-wide implementation of FRT to support gambling self-exclusion programs in the state of South Australia (SA). In 2022, the South Australian government enacted legislation that specified and determined the following:

“Any venues authorised to operate 30 or more gaming machines, if at least one machine can be operated using a banknote acceptor, must also have approved facial recognition technology installed in their gaming rooms. Facial recognition technology will support licenced venues meet their responsibilities of identifying barred patrons by alerting gaming venue staff when a barred patron is detected entering the gaming room” (SA Attorney-General’s Department 2021, n.p)

As one of the first jurisdiction-wide enforcements of FRT in small venue gambling, the South Australian scheme provides a test case for considering how the regulation came to be introduced and enacted, for what reasons, and with what outcomes.

Our study, therefore, pursues a number of different lines of inquiry. Alongside the eventual ‘on-the-ground’ experiences of gaming venue staff and self-excluded gamblers, we are also interested in the preceding interplay between the gaming industry, gaming regulators and IT industry actors in initiating this legislation. We are also interested in how, after many years of being judged *not* practically feasible, FRT is now being mandated in small gaming venues. For example, FR technologies continue to be criticised for impracticalities and inconsistencies when implemented in situ, especially in terms of processing images of non-white faces (see Buolamwini & Gebru, 2018). We are also interested in how the introduction of FRT in South Australian gaming venues is being justified in public discourse, and subsequently received amongst gambling-reform advocates, gambling industry bodies, and the wider general public. Indeed, the South Australia legislation was introduced during a period when FRT was prompting high-profile pushback with regards to its roll-out out into other sectors of society, with many civil rights commentators calling for heavy regulation or even outright banning of the technology in public places (Hartzog & Selinger, 2015, Australian Human Rights Commission 2021). All told, there is much to the application of FRT in SA gaming venues that merits further examination.

With these issues in mind, then, the remainder of this paper takes the South Australian FRT case and addresses the following research questions:

- How has facial recognition technology been framed as a necessary, legitimate and effective addition to SA gaming venues?
- What actors have been involved in advocating for/against FRT self-exclusion, on what grounds, and with what motivations?
- How does the technology-mediated exclusion scheme operate in practice?
- What are venue staff experiences of working with the technology in situ?
- How is the technology impacting the perceptions and practices of self-excluded patrons?
- What do key actors expect of the technology in the future?

The answers to these questions help provide some insight into the issues raised by both the use of facial recognition technology for limiting the harms of problem gambling, and the prospect of more widespread use of facial recognition technology for a growing range of purposes.

## Research Methods

These questions are addressed through analyses of empirical data generated as part of a wider five-year study into the ongoing integration of ‘smart camera’ technology into everyday domains of Australian society. The introduction and initial enactment of the SA legislation during the first two years of our study allowed us to follow the adoption of this technology as a real-time case study as events unfolded. While relatively non-contentious in comparison to discussions around the take-up of FRT by police and other law enforcement agencies, the issue of pokie regulation in South Australia still proved to be a controversial topic to research. Consequently, we found some officials and staff wary about commenting publicly on their work around the technology.

This meant that our own research draws on public statements and documentary analysis to gain a sense of industry and regulator perspectives, as well as information derived from off-the-record interviews with key officials. Elsewhere, we take care to not disclose specific names of venues and the specific FRT systems each has adopted in order to retain the confidentiality of the individuals that we did interview. Thus, we draw on the following data sources to explore the instigation and initiation of FRT into South Australian pokie venues from the perspective of gaming industry and IT industry spokespeople, government legislators and regulators, venue operators and staff, and a small contingent of self-excluded gamblers, gaming reformists and activists who represent the lived experience of gambling dimension:

- Interviews with SA government agencies, and with FR system suppliers.
- Documentary analyses of institutional statements, guidelines and policies drafted to support the implementation of FRT into gaming venues.
- Published interviews with government, regulator, IT and gaming industry spokespeople in news media and trade publications, along with documentary analyses, publicly-available news, social media and marketing materials relating to FRT companies whose products were approved suppliers to the South Australia scheme.
- Interviews with a mixture of self-excluded gamblers and those representing the lived experiences of this group and seeking reform of the gaming industry on these grounds.
- Visits to six venues of varying size (all with 30 machines or more) to observe how the venues (and enforcement of the FR scheme) operated.
- Interviews with 10 staff and managers in these six venues who were working with FRT in their venues.

Given the delimited nature of the case study, these empirical data are exploratory rather than generalisable – allowing us to highlight salient points that emerged during the rapidly unfolding roll-out of FRT in South Australian gaming venues. Our documentary analyses cover 2019 to 2022, while our interviews were conducted during 2022. Drawing on thematic analysis of this corpus of data, the paper explores the different ways in which the introduction of FRT into pokie venues progressed from the initial planning and development of the legislation through to its practical implementation in gaming venues.

Analysis of the empirical data was rooted both in the a priori concerns outlined in the previous section, and a posteriori issues arising organically from the documentary and interview data. In this sense, analysis took what Fereday and Muir-Cochrane (2006) describe as a ‘hybrid’ process of inductive and deductive thematic analysis that allows us to fully describe the phenomenon being investigated. This involved a number of steps. First, was the deductive generation of salient preliminary codes based on the six different areas of questioning and the different interest groups implicated in the self-exclusion program. We then engaged in repeated re-readings of the interview corpus, leading to the inductive generation of data-driven themes – i.e. issues arising from the interview data that we felt encapsulated the phenomenon of facial recognition-based self-exclusion enforcement as perceived by each different interest group. These themes are now discussed in turn for each of the following phases of the conception and implementation of the technology-based program, from abstract model to lived reality.

## Findings

(i) The origins of the FRT program

The South Australia changes arose from mounting political and public concern regarding the regulation of problem gambling. This followed a high-profile case from 2018 of a gambler who committed suicide after spending 13 hours playing the pokies, reinforced by subsequent estimates from the SA Coroners Court that one-in-ten suicides were linked to gambling addiction. These findings led to emerging media and political consensus that the existing self-exclusion program in SA was ineffectual. As one elected representative put it, “It was extraordinarily useless actually. And I think there was a recognition across the board from the welfare groups and the industry alike that that just didn’t work” [1]. Against this background, the idea of FRT being part of renewed efforts to help venues “deal with risky gambling” [2] arose toward the end of 2018, and was then hastened by the temporary enforced closure of gaming venues during the COVID shutdowns.

Public discussions at the time reflected an acknowledged need to strike a balance between “harm minimization” [2] and allowing the post-pandemic reopening of a local hotel industry employing more than 26,000 people. As the Attorney-General Vickie Chapman put it:

“Our gambling reforms have two key goals - support an important part of our economy and community, and ensure there are strong protections in place for vulnerable South Australians who find themselves needing support” [3]

The opportunity of addressing these goals then arose through hotel industry demands for the introduction of note-takers on poker machines in the State – i.e. allowing pokies in SA venues to accept banknotes as well as coins. Politicians in the two main parties seized on this request as an opportunity to leverage some regulation and governance initiatives – allowing them to claim to be addressing problem gambling while also being seen to support industry demands. As one local politician put it:

“Labour and Liberal did a deal to collude on passing that gambling reform. And it was a gambling reform that was at the behest of the [Hotels Association] and others around pokie machines for note dispensers. Basically, they were playing politics. So they wanted to make it look like they were coming up with harm minimisation measures. And so that’s where the facial recognition technology got put into the mix by [named politician] who’s now the treasurer. And it was a treasurer-to-treasurer deal. So obviously, the eye is on the money here” [1]

The integration of FRT was justified by local policymakers and gaming industry spokespeople as a low-risk and high-impact use. Venues in the state had already implemented ‘Automatic Risk Monitoring’ systems to identify persistent gambling in venues – as such FRT was positioned as “just another tool” [4] that could be deployed in gaming rooms. Facial recognition was already a familiar technology to the gaming industry, with large casinos in many Australian state capital cities having been required to introduce the technology. FR industry interviewees reported having lobbied local gaming stakeholders prior to the state government’s proposals: “we spent a lot of time with the hotels association, Clubs SA … educating people about how facial recognition could be used and … telling them how they could take advantage of the system basically and how they could use it and apply it to their requirements” [5]. As such, much of the groundwork had already been done to develop local gaming industry awareness and enthusiasm for FRT - both as a means to respond to the perceived fallibilities of the human-operated self-exclusion system, as well in immediate terms of pushing through the introduction of note acceptors as well as longer-term potential uses for customer profiling and savings in labour costs.

In addition, the SA government was also keen to position itself as technologically advanced – as one FR industry interviewee put it: “South Australia likes to be seen as the trailblazers in anything to do with technology … they just like to be seen as the leaders” [5]. The South Australian police had already been using FRT in Adelaide’s CBD since 2019, and the SA Health authority had trialled a FR app to enforce home quarantine during COVID. In addition, Adelaide is considered a hub of biometrics research in Australia through its local Defence, Science and Technology laboratory and two of the city’s research universities. In this sense, introducing FR self-exclusion in gaming venues was welcomed by claims of “an Australian first” [6] and further symbolised a state government “always considering new and emerging technologies and what protections and safeguards are in place” [7]. In this sense, there were various and important symbolic, economic and operational dividends closely tied to the introduction and roll out of FRT in gaming venues. It was framed as a vehicle that could deliver a multitude of different benefits both to the image and management of gambling practices in South Australia.

(ii) Negotiating the details

Against this background, came the introduction of what was reported in news media as “sweeping gambling reforms”. Government permission for note-taking machines was accompanied by various harm reduction measures proposed by Labor politicians – including the stipulation that:

“clubs with more than 30 poker machines, where any of those machines are able to accept bank notes, must use facial-recognition technology to help detect people who have barred themselves or have been barred due to issues associated with gambling-related harms’ [8]

**Fig. 1:**
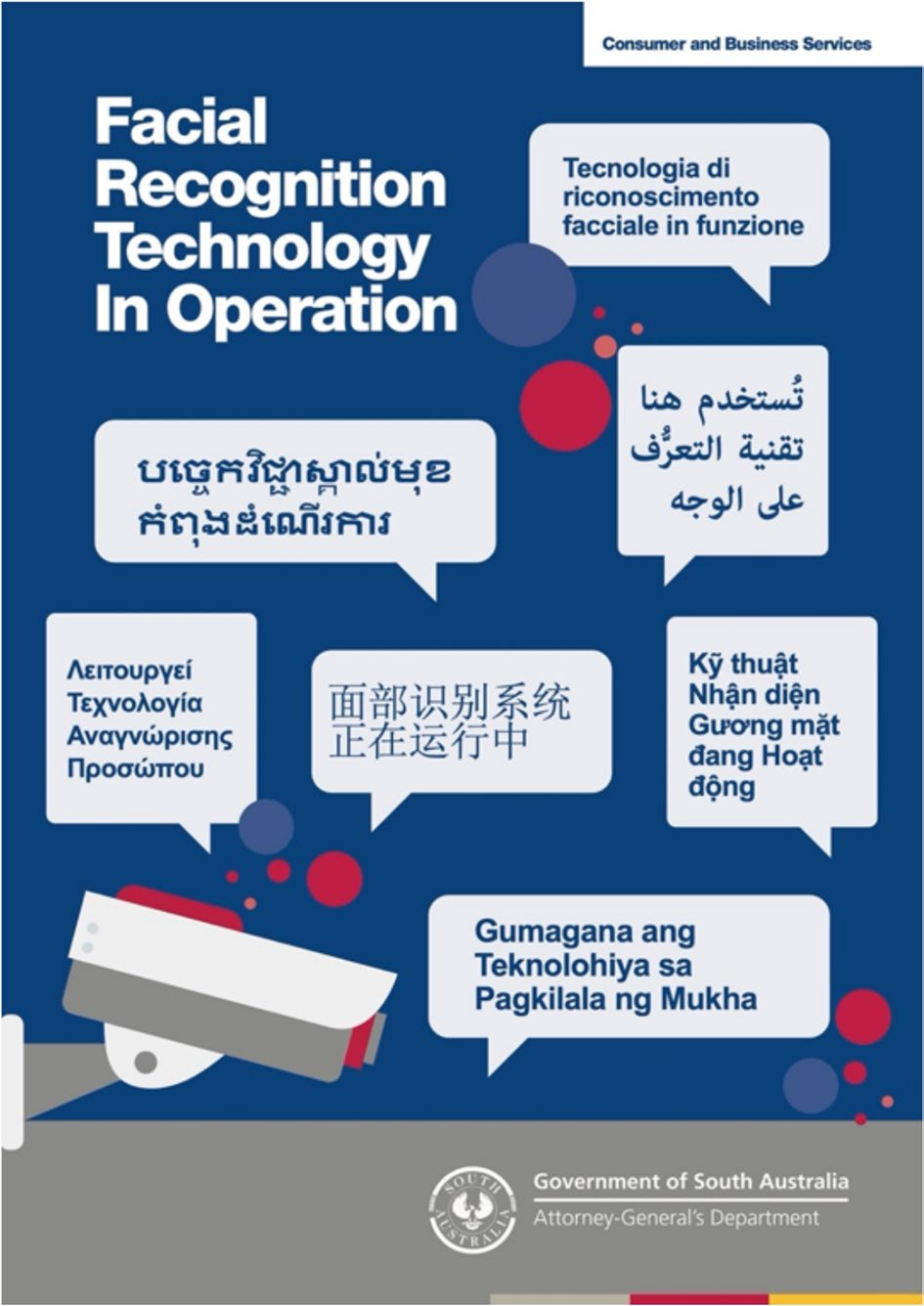
Multilingual signage designed to be deployed in all participating game venues

This meant that in excess of 230 venues were then required to install FRT systems – a boon for vendors. Each venue was required to procure and install one of seven different mandated systems from six FR vendors – five based in Australasia and one in the US. These systems were to be accompanied by prominent signage in each of eight languages spoken by local communities (see Fig. 1), with a small team of inspectors tasked with ensuring compliance and “regularly checking venues to ensure that camera placement is optimal and detections are occurring” [9].

These arrangements had been subject to various negotiations between government officials and hotel industry groups – including discussions over whether signs should be displayed at the entrance or within each venue, where cameras should be located, and the acceptable levels of face-matching accuracy. These negotiations reflected the subtly different standpoints of various stakeholders. For example, one government official (clearly not wanting to be seen to be encouraging any form of gambling) recalled rebuffing suggestions from the hotel industry to “put the cameras up next to the jackpot number, because everyone looks up at that to see … well, yeah, sorry.” [4].

All told, most of the stakeholders appeared to be satisfied with the eventual arrangements. The hotel industry were also content with the choice of different technology providers—“[it] creates a level of competition and makes [the system providers] more price-competitive” [10], even though a couple of the local FR firms “took a very big percentage of the market early on” [4]. The state government and politicians could also claim to be intervening in the issues of problem gambling. As one national newspaper editorial contended:

“It promises to be a hard-fought battle but action is necessary. The government should be credited for taking on the powerful voices of the clubs and pubs industry in this way, because problem gambling in this state needs to be stopped before it ruins any more lives” [11].

That said, this implementation of FRT was not unanimously accepted. Anti-gambling groups remained sceptical of the trade-off between harm reduction and the introduction of banknote-acceptors: “this will just make it even easier for people to get into trouble” [12]. As another gambling reform campaigner put it: “don’t bother about facial recognition … [just] take the addictive nature of the machines away!” [13]. Alternately, some opposition politicians raised concerns over privacy and personal freedoms. For example, council members in the SA capital city of Adelaide saw the incursion of FRT into small pubs and hotels as raising “an issue of proportionality” in relation to other more valued uses of the technology such as “tracking the movements of people like terrorists” [14]. As a prominent politician from another state reasoned:

“I don't support facial recognition technology. It is too Orwellian. It will scare people and it is unnecessary. You don't need facial features checked when you walk into a pub” [15].

Such concerns raise the more general issue of the normalisation of the use of facial recognition technology for a growing range of purposes in commercial venues and public spaces.

(iii) The realities of the roll-out

Across 2021 and 2022, the program soon settled into venues’ routine operations, with authorities heralding the scale and success of enforcement. In the first six-months of the program, authorities were claiming “more than 50 million faces have been scanned using facial-recognition technology in South Australia, with more than 1700 detections of potentially barred patrons” [8]. A year later we were informed that around 367 million face scans had been conducted, leading to over 9000 ‘hits’ on possibly excluded individuals [4]. As one key official told us, despite the initial unease, the program was proving to be welcome change for gambling venues, thanks in part to the introduction of note collectors, which make it easier for patrons to spend greater amounts of money more quickly.

“to be honest, this went in surprisingly quietly. Everyone, I think, realized how much money they're gonna make from facility and care. So that's proven right. So we've had an uplift in gambling revenue to venues in the state since introduction of [note] acceptance” [4]

Indeed, it was widely felt that the introduction of FRT had not impinged on venue gaming profits, leaving FR industry spokespeople confident to point to evidence of the effectiveness of their systems:

“a lot of the clubs that we have using our system in have seen remarkable results since when the system was put in. … as an example, there’s one venue and they thought they might intercept a self-excluded gambler maybe four or five times a month, … after they put our facial recognition system in, the numbers were more like 30 to 40 a month. So massive, massive differences … the results that people are getting and talking to us about – it’s all very positive stuff – it’s quite remarkable. It makes you wonder how that manual system operated” [5]

Our own in-person visits to venues revealed a more mixed picture. From a positive point of view, all of the six venues we visited with 30 machines or more were complying with the scheme requirements for prominent notifications about the use of facial recognition posted on walls and entrances. In most venues, FR system screens and consoles were kept hidden from customers as required (although one venue had a computer screen that was clearly visible by customers, which they were happy to show us and to demonstrate how it operated). Indeed, regardless of venue size, staff and managers were largely in agreement that the new scheme was ‘working better’ [16] than the previous reliance on manual recall of faces. Although a few patrons would now self-consciously hide their faces when entering the venues (pulling hats down, wearing sunglasses), it was felt that most did not notice the presence of the cameras, screens and posters. They had become, for all intents and purposes, normalised and naturalised as background infrastructures in what is designed to be a highly stimulating environment. As one manager put it, “all they are interested in doing is putting coins into the machines – they don’t really notice anything else” [17]. Staff suspected that gamblers who knew they were on the self-excluded list would be likely to stay away from venues that have automated facial recognition and, instead, seek out smaller camera-less venues under the 30 machine limit. Obscuring one’s face was not a workaround for excluded gamblers, because staff are required to ask them to remove hats or hoods that might conceal faces from the cameras or distort how faces come to be algorithmically read and profiled.

However, in contrast to the notion of automated enforcement, venue staff were keen to point out that the FRT scheme did not reduce their workload or responsibility in any significant way. One staff member bemoaned the stress caused by their FR system making a positive match. As we were shown, once the system detects a possible match, an alert appears on the attendant’s computer screen, and staff are required to complete a report confirming that either: (i) the match was positive and the person has been reported and expelled; or that (ii) the match was a false positive [18]. Given the time pressure of the job (many small venues had just one staff member covering the pub, restaurant and pokie areas), some staff had taken to giving people five seconds to leave as soon as the system reported a match – warning them that otherwise the venue was subject to a fine of up to $10,000. In this way, the technology was enrolled as an external arbitrator of un/belonging in the venue, with a complex set of socio-technical relations mediating and determining the outcome of a face match being made. Far from relieving venue staff of supervisory work, these arrangements placed a heavy burden on them to perform a novel form of emotional labour as they confronted ‘suspect’ individuals rightly or wrongly recognised by the machinic gaze in different ways, and acted as boundary maintainers by either accepting or refusing their entry. Moreover, the FRT system was felt to place further accountability onto them, as often casual and precarious workers, to ensure moral and legalistic processes were adhered to and actioned.

That said, detection rates were not felt to be high. Staff in smaller venues reported getting a ‘few matches a week’ [16], with these often turning out to be false positives. Indeed, staff in all venues noted the fallibility of the technology. One venue noted a tendency for the misrecognition of gender (women being mis-identified as men), and their system’s reduced accuracy at night-times. Another bemoaned the repeated misrecognition of particular individuals over time, leading to the awkward shared acknowledgement between staff and a few regular customers that the system would be erroneously triggered whenever they visit. The manager of one venue in the port district of Adelaide (an area of relative social disadvantage) reported their system’s propensity to generate more errors – in this case false positives – for Indigenous customers.

Despite these glitches, our interviews with self-excluded gamblers also conveyed a broad sense of qualified approval. All the self-excluded gamblers we spoke to welcomed any effort to bolster the program by adding, “An extra layer of protection if you are tempted to go into the venue knowing that it’s there” [19]. The presence of FRT was seen to further deter individuals who were struggling to maintain adherence to self-exclusion orders. For example, one interviewee – describing herself as “totally paranoid about being found out and being done under the self-exclusion laws” – took the presence of FRT as a sign that the self-exclusion program would be enforced and that she would likely be identified: “If I thought that was going to happen for one second, there’s no way I’d go near it” [19]. Although there can be fines issued both for venues and self-excluded gamblers for violations of the ban, enforcement remained variable. Our discussions with officials involved in the program indicated they were more interested in ensuring excluded gamblers were removed from the venues than in levying heavy fines. Nevertheless, for some, the prospect of being identified was heightened by the presumed technological capabilities of FRT. The act of having one’s face remotely and automatically scanned and checked was felt to be a more visceral ‘precise’ act of identification: “Definitely, yeah. This is going beyond just giving your ID that has a static photo and your details. This is a much more realistic and, what’s the word, just precise capture of your face” [20]. In the classic mould of routine activity theory and capable guardianship, the technology, in other words, was perceived to add a veneer of objectivity, accuracy and efficacy to the management of facial profiling, and the very knowledge of its operation, however in/effective it may be in practice, acted as a notable deterrent.

Nevertheless, the self-excluded gamblers we interviewed regarded the frailties of how the FRT was acted on by venue staff after any technological identification had been triggered. A degree of scepticism remained over the enforcement of the new arrangements:

It’s good, as long as it’s not tokenistic. I think it needs to be properly implemented, people need to be properly trained to use it and venues need to properly staff it as well. It’s all very well to walk past a camera and a picture of your face is taken, but if nothing’s done with that, it could be a false sense of security [19]

I support it with the provisos that the person monitoring knows how to handle somebody. And that’s the only reason I would support it, otherwise I think it could be abused in retrospect. Like, I know [prior to the FRT scheme] a friend of mine went in and played at a venue, and he was on the self-exclusion list. And they were okay, let him play – but the minute he won some money and needed a cheque they said, “No, we can’t give it to you.” … It’s a trust system, isn’t it? And I don’t know about you, but I don’t trust venues. [13]

The scenario described here would be illegal, but any complaint on the part of the self-excluded gambler would require admitting the initial violation of entering the venue. Such observations nevertheless reflect a knowing cynicism toward the industry that came across in a range of interviews with customers as well as activists and public officials.

(iv) Settling down and looking forward

While our study covers only the first couple of years of this program’s development and implementation, at the time of writing there are few signs of any appetite for its curtailment. In contrast, we found increasing acceptance of the system from venues and patrons alike, with FR industry and policymakers eager to push for the continued expansion of the program. As one industry spokesperson put it, the gambling ‘use case’ provided a public manifestation of how FR technology could be of clear social benefit:

“I don’t think we’ve come across anyone that’s against it. … once the systems went in and they saw how easy it was to operate and run – like honestly they don’t need to do very much at all … [In Australia] I feel that it’s becoming a lot more accepted by the public, because people are seeing the benefits of the technology. It’s not like we’re a communist country where we’re looking for specific minorities and tracking them. Our customers are using it for very legitimate reasons that help not just their staff but the community. … I think people are starting to see that in public. So I think it's becoming more widely accepted around Australia” [5]

Tellingly, we also found a normalisation of the technology’s fallibility. Government officials reported most venue managers having settled on setting system thresholds around 70 percent for triggering the alert for identifying banned patrons (i.e. the system being 70 percent certain of any match) – “obviously, if you want to set it to 99.9, you probably never pick up anybody. … so in the 70s [seems] about the mark where it's pretty sure it's the right person” [4]. While this configuration of venue systems was obviously resulting in mis-identifications, this was considered acceptable by FR industry representatives:

“you do get false positives but we’re not convicting anyone. We just want to have a discussion with someone to make sure that they are the right person and we want to make sure they want to be where they want to be if they’ve asked for help” [5]

Indeed, industry and government officials were keen to imagine the expansion of the technology – for example, identifying gamblers who were lingering in venues for excessive periods of time, enforcing liquor licenses, and even flagging “whether or not somebody's had a good experience within a venue” [4]. Moreover, at the time of writing, the South Australian program is beginning to prompt discussions of being replicated in other states, leading to headlines such as “Facial recognition system could be rolled out to pokies rooms across Australia” [21]. As politicians and hotel industry representatives in New South Wales put it:

“Pokie machine addiction is an old reality, and we have the technological solution to help which is what we must do when someone asks for it because it's destroying their life” [22]

“Those that have been proactive in choosing to self-exclude from the gaming rooms of clubs and pubs will now have extra support from our industry to make sure they maintain their resolve and stay out of harm’s way [23]

Thus, in contrast to the initial ‘Orwellian’ concerns noted previously, the SA case is now being used to support arguments around the need to further implement FRT on a nationwide basis. Indeed, during our interviews, such rationales were challenged only by the self-excluded gamblers themselves:

“I think it looks really good if something like this is rolled out. It looks great for politicians if they can implement this successfully… I think that the gambling industry is too big and too profitable for it to ever stop the juggernaut that it’s on at the moment, but I think politicians have realised that they’ve got to do something, or appear to be doing something, about problem gambling. Facial recognition technology’s a really easy win if you’re a politician, to get that across the line and look good. I think that’s why it was picked up”. [19]

## Discussion

At first glance, these findings depict a relatively straightforward implementation of a technologically-enhanced self-exclusion program. While FRT might have initially be seen as a controversial technology, it seems to have become quickly established as a routine addition to small gaming venues. Venue staff were in agreement that the system is an improvement on manual checking, venue owners were profiting from adding note-taking capabilities to their machines, politicians could tout their concern for addressing problem gambling, and even self-excluded gamblers could see exclusion programmes being given a little more (technologically-augmented) rigour. As such, there is every reason to anticipate this technology continuing to be taken-up over the next few years, and becoming an integral element of gaming provision in many similar jurisdictions. However, the introduction of facial recognition into small gaming venues in this manner is *not* simply a straightforward technological solution to reducing the widespread harms caused by the industry and the pathological forms of behaviour its infrastructures and cultures cultivate. Instead, the developments in SA as described in our study could be described as a tangle of motivations and political manoeuvring that potentially introduces a set of new risks and possibly new harms. As such, a number of points for further discussion arise.

First, despite the confident claims of system vendors and government officials, this does not seem to be technology that offers a perfect solution to the frailties of the previous modes of self-exclusion. In comparison to the use of FRT in the relatively controlled environs of other contexts such as airports, gaming venues are clearly unconducive conditions for accurate face-matching. Settling on a threshold of around 70%, meant that the technology was producing high levels of erroneous matches (and presumably also regular instances of *not* matching people that should be identified). The operation of the system was still reliant on the discretion of venue staff and, if anything, it appeared to be increasing work for staff having to deal with the social frictions arising from the prevalence of false-positives and repeat errors. As such, we found venue staff and self-excluded gamblers continuing to work with – and work around – what remained an inconsistent process. Elsewhere, the wider population of problem gamblers who had *not* opted to self-exclude were now subjected to the exponential risks arising from note-taking machines being introduced into venues, while local politicians could claim to have initiated high-tech hard hitting reforms. In this sense, FRT seemed to be more of a symbolic gesture than an impactful intervention. It also served to defer the implementation of potentially more effective solutions, such as cashless cards with set “’pre-commitment’ limits that allow punters to decide before they enter a venue how much they can afford to lose – rather than being caught up in contextual conditions built around the principle of “addiction by design” (Schull, 2012).

Thus, any sense of ‘benefit’ of the FRT scheme is clearly dependent on context and circumstances. Clearly, the installation of FRT in gaming venues is seen to have ‘worked’ from the point of view of gambling regulators and the hotel industry. Moreover, we certainly found FRT to be associated with an increased sense of certainty – i.e. the presence of cameras allowing venues to signal their compliance to the scheme, and signalling to the self-excluded that the system might be better enforced. However, we also found instances where the technology was not ‘working’ for others – not least the higher propensity for Indigenous customers to be misidentified in one venue, or a few regular customers in another.

How the gaming community responds to these instances of system failure is of key importance. From a technical point of view, instances of error and mis-recognition are a feature of all forms of FRT. Occasional misrecognition is to be expected in gaming venues, which lack the ideal conditions for the cameras to function (high levels of illumination, head-height cameras fully-facing the customer). Thus, as one of our industry interviewees put it, such errors are to be tolerated (“you do get false positives but we’re not convicting anyone”). Yet, while reported levels of ‘false positives’ and ‘false negatives’ might remain acceptable in statistical terms, over time, they still involve a significant number of people being erroneously ‘recognised’ by these systems in real-life. Whether or not one is perturbed by not being allowed into a gaming venue probably depends on how often this inconvenience occurs – and what its consequences are.

In particular, the sense that these venue systems did not appear to be working well with Indigenous or female customers chimes with the wider literature on the adverse impacts of facial recognition in society – where trial programs and test-cases continue to show the propensity of FRT to mis-recognise certain groups of people more frequently than others. In particular, evaluations of FRT continue to show racial bias, and a particular propensity to mis-recognise women of colour (see Buolamwini & Gebru, 2018). This raises the problem that the nature and extent of these FRT system failures in small gaming venues is being experienced disproportionately – with already minoritized populations bearing the worst effects. Such observations also highlight the importance of paying close attention to how the installation of FR technology that many people might be experiencing as unproblematic might nevertheless be “encod[ing] systematic inequalities” (Benjamin, 2020, p.3). In other words, more attention needs to be paid to how the design, development and implementation of FRT can nevertheless “often adopt the default norms and power structures of society” (Benjamin, 2020, p.3) and therefore end up disadvantaging already marginalised population groups.

Finally, given these issues, a worrying point of contention to arise from our interviews was the possibility of FRT being adopted for an ever-expanding range of purposes in gaming venues (and beyond) – what might be described as processes of ‘function creep’. The argument here is that even ostensibly benign implementations of FRT introduce logics of automated monitoring, tracking, sorting and blocking into everyday public and private spaces that can then lead quickly onto further (and initially unanticipated) applications – what Andrejevic (2020) describes as a ‘cascading logic’ of automation. For example, scanning the faces of gaming venue customers to identify self-excluded problem gamblers in real time may seem like a virtuous use of the technology. Yet the introduction of this technology fits with other uses that venue owners and marketers might also welcome. Slot machine manufacturers are already using FRT in machines to recognise repeat customers and personalise advertising and gaming experiences. This logic can then easily be extended into recognising (and deterring) repeat customers who spend only small amounts of money or whose appearance is not in keeping with the desired aesthetic of the venue. Given that this technology was primarily justified to South Australian venue owners under the guise of allowing them to increase profits by fitting note-taking machines, further moves beyond initial intentions of harm reduction would be understandable. This is clearly a case for oversight and regulation.

## Conclusions

All told, while the introduction of FRT into gaming venues might appear to offer a relatively straightforward and benign improvement to the efficiency of the SA self-excluded gambler program, in many ways, the technology is associated with heightened inconsistencies, inefficiencies and uncertainties. For example, the FRT-driven program does not appear to better address the core issues underpinning problem gambling. Despite industry and government claims to the contrary, these developments seem unlikely to substantially improve conditions for problem gamblers to operate within. The technology is also associated with added uncertainties – not least the ways in which its application might extend and expand in the future. As such, the gambling sector needs to pay close attention to how initial cases such as South Australia continue to unfold ‘on the ground’, and perhaps refrain from wider replication of this technology in other jurisdictions. Regardless of the enthusiasms of government, tech industry and gaming lobby, FRT is certainly not a straightforward technical fix to problem gambling.

## Data Availability

Anonymised versions of the interview dataset of the current study is available from the corresponding author upon reasonable request. All documentary sources are publicly available.
